# A geroscience mouse model for Alzheimer’s disease

**DOI:** 10.1080/20010001.2019.1616994

**Published:** 2019-05-14

**Authors:** Martin Darvas, Dirk Keene, Warren Ladiges

**Affiliations:** Departments of Pathology, University of Washington, Seattle, WA, USA; Comparative Medicine, School of Medicine, University of Washington, Seattle, WA, USAwladiges@u.washington.edu

Geroscience is a multidisciplinary field that examines the relationship between biological aging and age-related diseases [1]. Seven processes discussed by the trans-NIH Geroscience Interest Group Summit that contribute to biological aging included macromolecular damage, epigenetic changes, inflammation, adaptation to stress, impairments to proteostasis, stem cell regeneration, and metabolism [2]. These processes are highly integrated with one another such that targeting them as a group may be an effective approach to developing therapies to prevent or delay age-related disease.

Alzheimer’s disease (AD) is an age-related disease and is expected to increase with the number of elderly individuals rapidly rising in both developed and developing countries. Efforts to find disease-modifying treatments have met with limited success possibly because they have focused on identifying a specific pathogenic mechanism targeted by a specific drug. AD is a complex disease involving numerous mechanisms in line with processes of biological aging. Therefore, a geroscience approach to successfully treating AD is a logical concept that unfortunately has not yet been widely accepted by the neuroscience community. It is now time to explore preclinical studies in AD animal models to begin screening different drug combinations that target multiple aging-related processes for effect on AD dementia and neuropathology.

**Figure 1.**
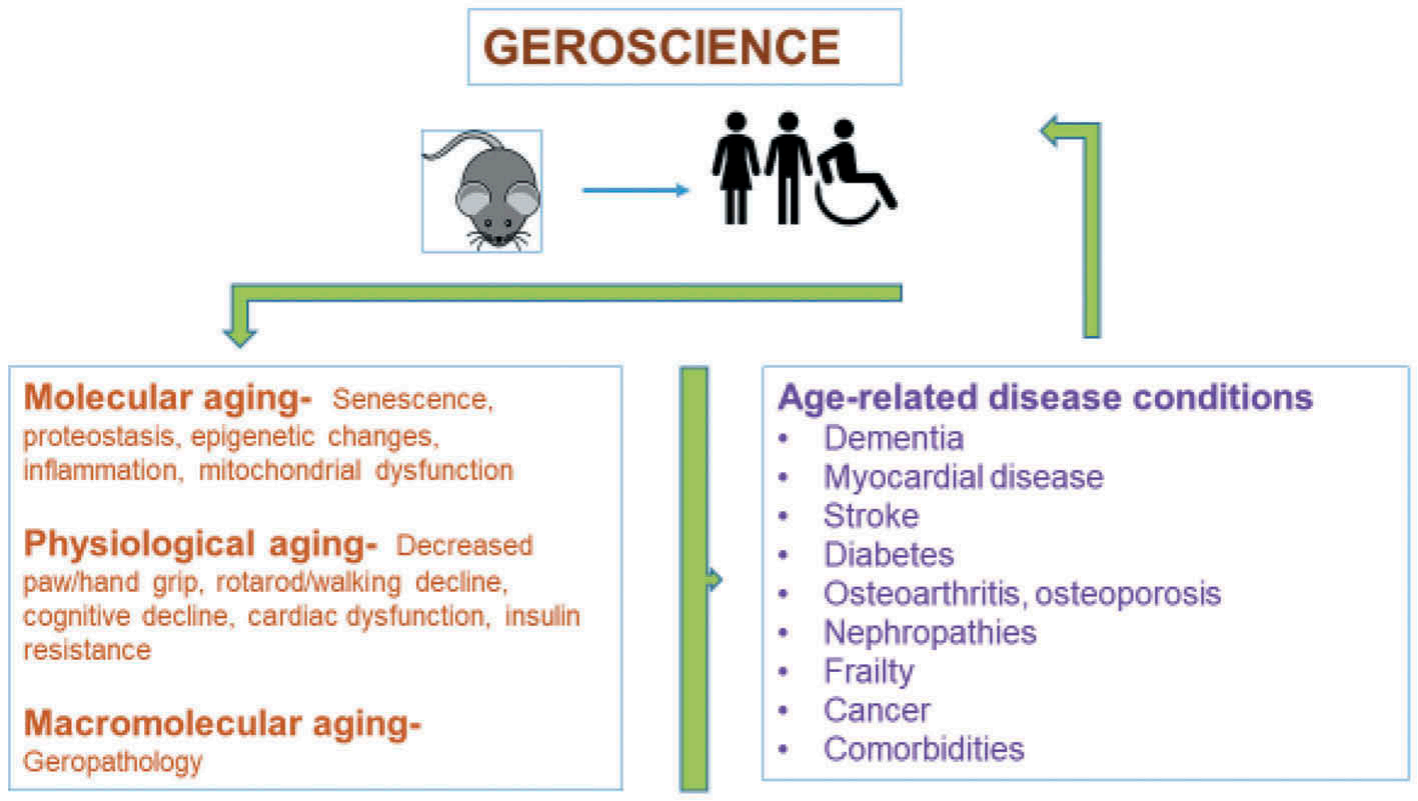
The relationship between biological aging and age-related diseases can be studied as a multidisciplinary field known as geroscience. Aging mice can be used as models of human aging to investigate the effect of aging on age-related diseases.

A major challenge for preclinical drug testing is the selection of an AD animal model. A model is needed that shows amyloid (A) β and tau neuropathology, inflammation, oxidative stress, neuronal degeneration, and neurovascular deficits in an aging background. Currently available models are transgenic mice expressing amyloid precursor protein (APP) and presenilin mutations found in patients with early onset of AD. These mouse models are useful, but develop lesions at an early age, and none represent all the mechanisms representative of human AD. Ideally, the model should be easily manipulated so that dementia and neuropathology can be induced in an old-age animal as well as middle age, and young age to compare disease progression in different aging backgrounds. The animal of choice for large-scale drug testing is the mouse, but the rat could also be considered. There are advantages and disadvantages for both but our lab has extensive experience with aging mice so the aging mouse will be the prototype animal for this discussion. Aging in mice is in many ways similar to aging in people, so the geroscience concept is applicable, as depicted in Figure 1.

The two hall-mark molecular pathologic changes of AD are accumulation of amyloid β 42 peptides (Aβ42) and paired helical filament (PHF)-tau (τ). Aβ42 pathology is not evenly distributed, but systematically localized to certain parts of the brain, following a prototypical sequence in which the regions are hierarchically involved: cortical Aβ42 deposits, followed by involvement of allocortical regions, involvement of subcortical forebrain regions and striatum, deposits in the brainstem and finally cerebellar Aβ42-deposition. The distribution of Aβ42 across the brain is part of the current NIA-AA consensus criteria for the neuropathologic diagnosis of AD [3]. To allow control over the extent of Aβ42 expression and better alignment with the clinical distribution of Aβ42, an AD model that is based on adeno-associated virus (AAV) mediated Aβ42 expression was developed and validated in adult rats [4]. This model overcomes the shortcomings of current transgenic models of AD pathology because it allows induction of pathology at a disease-relevant age and restricts pathology to injected brain regions selected based on their relevance to AD.

**Figure 2.**
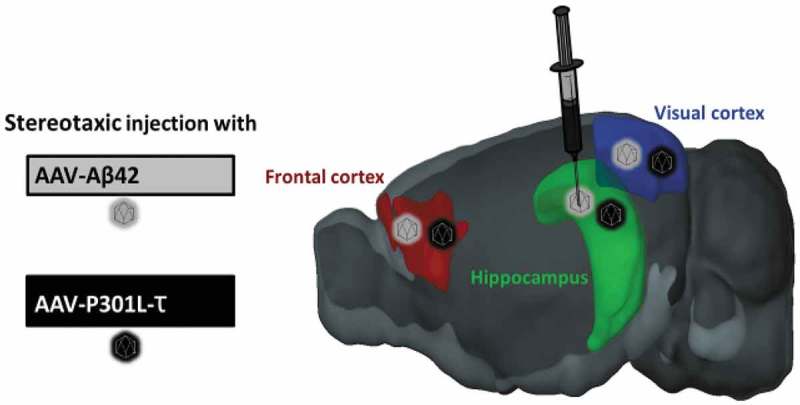
Multiple brain regions of the aging mouse can be targeted with Aβ42 and P301Ltau. This allows induction of pathology at a disease-relevant age and restricts pathology to injected brain regions based on their relevance to AD.

While genetic data clearly support mutations in the *APP* gene as sufficient to produce AD, and extensive experimental evidence points to the neurotoxicity of Aβ peptides, it is now appreciated that cerebral cortical accumulation of fibrillar Aβ occurs in virtually all older adults and reaches maximal concentration in the pre-clinical or prodromal stages of AD prior to clinical expression of dementia. In contrast, AD dementia is closely associated with the extension of PHF-τ beyond mesial temporal lobe structures. The distribution of PHF-τ pathology in AD also follows a prototypical sequence with hierarchical involvement of the following regions: transentorhinal cortical layer, entorhinal layer and hippocampus, and isocortical PHF-τ containing tangles. Similar to Aβ42, the distribution pattern of PHF-τ is part of the current NIA-AA consensus criteria for the neuropathologic diagnosis of AD [3]. Therefore, we also include the formation of PHF-τ in AAV-based models of AD. Although there are no known associations between mutations in the gene encoding τ (*MAPT*) and AD, there are strong associations between mutations in *MAPT* and hereditary frontotemporal dementia and Parkinsonism [5]. One such mutation (P301L) results in PHF-τ accumulation and has been previously used to develop an AAV-mediated tauopathy model in rodents [6]. Therefore, the ultimate AAV-based model of AD is to use stereotaxic injections of two AAVs (AAV- Aβ42 and AAV-P301L-τ) into multiple brain regions including cortical (e.g. frontal cortex) regions and the hippocampus (Figure 2).

To better understand the relationship between aging and AD this model can be applied to middle age mice (e.g. 12–14 months) and to old age mice (>20 months) with existing age-related disease conditions, thus covering the spectrum of geroscience. Increased knowledge regarding biological aging into research on AD will provide a more predictive venue for developing new and successful drug treatment regimens for AD.
